# Nomograms to predict the prognosis in malignant ovarian germ cell tumors: a large cohort study

**DOI:** 10.1186/s12885-022-09324-7

**Published:** 2022-03-10

**Authors:** Zixuan Song, Yizi Wang, Yangzi Zhou, Dandan Zhang

**Affiliations:** Department of Obstetrics and Gynecology, Shengjing Hospital of China Medical University, Shenyang, 110004 People’s Republic of China

**Keywords:** Malignant ovarian germ cell tumor, Nomograms, SEER database, Prognosis, Overall survival

## Abstract

**Background:**

Malignant ovarian germ cell tumors (MOGCTs) are rare gynecologic neoplasms. The use of nomograms that are based on various clinical indicators to predict the prognosis of MOGCTs are currently lacking.

**Methods:**

Clinical and demographic information of patients with MOGCT recorded between 2004 and 2015 were obtained from the Surveillance, Epidemiology, and End Results database, and Cox regression analysis was performed to screen for important independent prognostic factors. Prognostic factors were used to construct predictive calculational charts for 1-year, 3-year, and 5-year overall survival (OS). The externally validated case cohort included a total of 121 MOGCT patients whose data were recorded from 2008 to 2019 from the database of the Shengjing Hospital of China Medical University.

**Results:**

A total of 1401 patients with MOGCT were recruited for the study. A nomogram was used to forecast the 1-year, 3-year, and 5-year OS using data pertaining to age, International Federation of Gynecology and Obstetrics (FIGO) staging, histological subtype and grade, and surgical type. Nomograms have a more accurate predictive ability and clinical utility than FIGO staging alone. Internal and external validation also demonstrated satisfactory consistency between projected and actual OS.

**Conclusions:**

A nomogram constructed using multiple clinical indicators provided a more accurate prognosis than FIGO staging alone. This nomogram may assist clinicians in identifying patients who are at increased risk, thus implementing individualized treatment regimens.

## Background

Malignant ovarian germ cell tumors (MOGCTs) constitute approximately 1–2% of all ovarian malignant tumors with a predilection to the younger age group, especially during late adolescence and young adulthood [1, 2]. MOGCTs mainly include dysgerminomas, yolk sac tumors, teratocarcinomas, non-gestational choriocarcinomas, and mixed MOGCTs containing at least two types of malignant tissue [3]. Due to the sensitivity of MOGCTs to chemotherapy, most patients undergo fertility preservation surgeries [4]. The prognosis is usually good, with a 5-year overall survival (OS) of 95% for stage I tumors and 73% for advanced stage II–IV tumors [5]. Mangili et al. showed that the OS of patients with MOGCTs is correlated to tumor stage and histological classification, but not surgical type, tumor size, or tumor marker elevation [5]. Newton et al. also determined that histology has a significant effect on prognosis [6]. However, the risk factors for OS in patients with MOGCTs have not been evaluated in a large multicenter cohort.

In the current study, the incidence of tumor and survival data of approximately 34.6% of all cancers in the US were collected from the Linked Surveillance, Epidemiology, and End Results (SEER) database (https://seer.cancer.gov/), which is a reliable cancer information source [7]. A study based on the SEER database has the advantage of targeting a larger population from different geographical areas compared with a single-center study. The nomogram scores of individual disease-related risk factors can be calculated and used to predict prognosis. In recent years, gynecologists have begun to acknowledge it as an applicable tool [8, 9]. However, there is a lack of research on the construction of a visualized nomogram for MOGCTs. In this study, a nomogram was constructed to predict MOGCT survival using a cohort based on the SEER database of patients with MOGCT and correspondingly assess factors associated with OS.

## Methods

### Ethics statement

It is not compulsory to obtain informed consent from patients regarding the use of the SEER database as cancer cases are reported in all states in the United States. This study followed the 1964 Helsinki Declaration and subsequent amendments or similar ethical standards. This retrospective study included MOGCT patients from 2008 to 2019 in Shengjing Hospital of China Medical University and was approved by the Ethics Committee of the hospital (Ethics Code: 2020PS814K).

### Patients

**Fig. 1:**
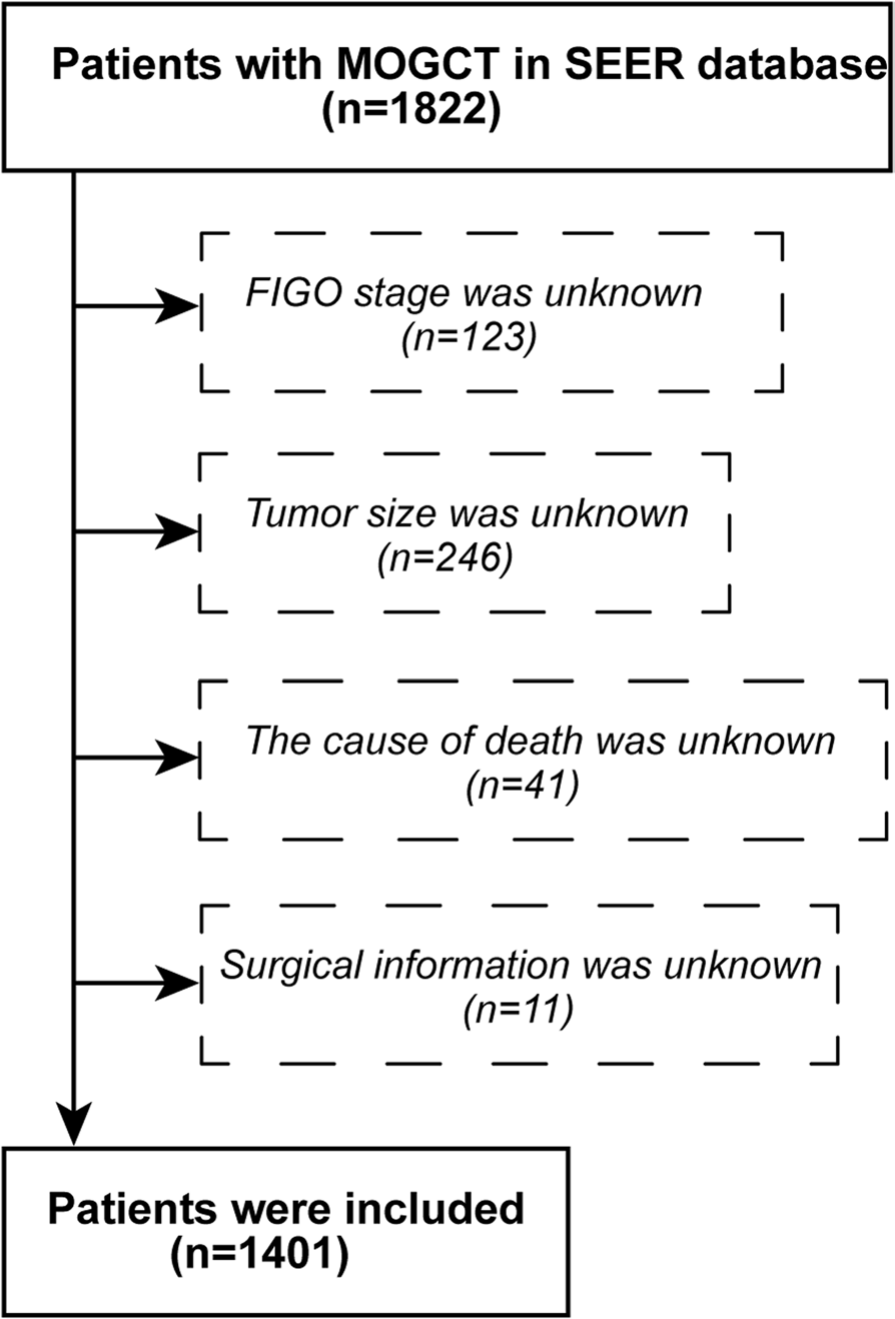
Flow diagram of patient selection criteria

Data of MOGCT patients registered between 2004 and 2015 were collected from the SEER database using SEER*Stat version 8.3.6.1. The locus code was C56.9, and the histological code was 9060/3–9110/3, according to the International Classification of Tumor Diseases, 3rd Edition (ICD-O-3). The exclusion criteria included: (1) unrecorded Federation International of Gynecology and Obstetrics (FIGO) stage, (2) unrecorded cause of death, (3) unrecorded tumor size, and (4) unrecorded specific surgical methods. The externally validated case cohort included a total of 121 MOGCT patients from 2008 to 2019 from the database of the Shengjing Hospital of China Medical University. A patient selection criteria flow chart is shown in Fig. 1.

### Data collection

**Fig. 2:**
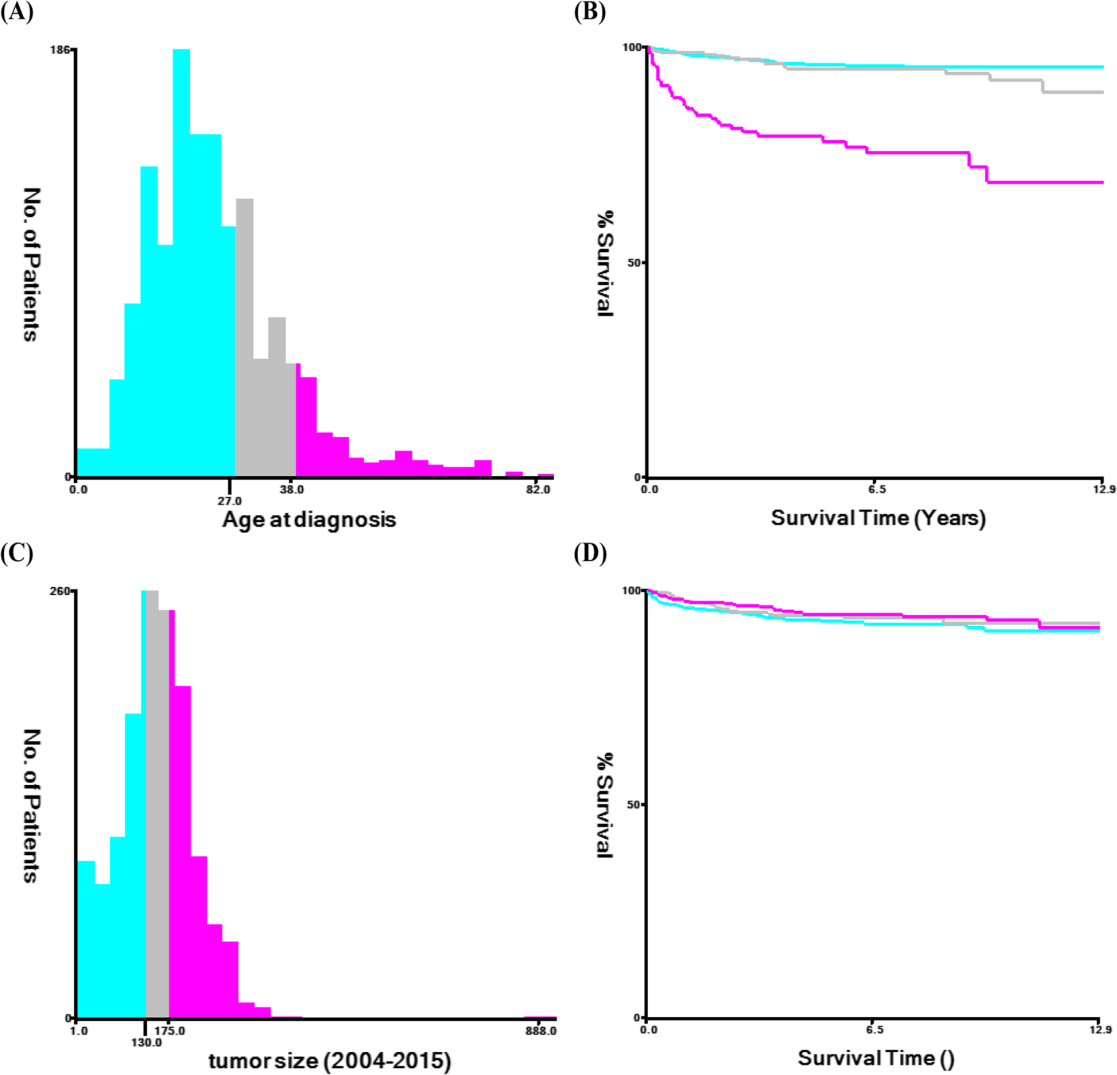
The thresholds for age and tumor sizes were established by X-tile analysis. (**A**, **B**): The thresholds for age were 27 and 38 years; (**C**, **D**): The cutoff values for sizes of tumor were 130 mm and 175 mm

Patient information was obtained from the SEER database, including patient ID, age, size of tumors, FIGO staging, laterality, histological subtype and grade, surgery, radiotherapy or chemotherapy, survival time, survival status, and cause of death. X-tile software [10] was used to evaluate the suitable thresholds for patient age and tumor size (Fig. 2), which were 27 and 38 years and 130 mm and 175 mm, respectively. The duration from the beginning of treatment to death or the last follow-up appointment was considered as the OS.

### Statistical analysis

Optimal thresholds for tumor size and patient age were established using the X-tile software. The data was analyzed in the RStudio environment using R (version 3.6.3; R Foundation for Statistical Computing, Vienna, Austria; http://www.r-project.org). To assess elements correlated with independent survival, univariate and multivariate Cox regression analyses of our clinical data were conducted. Hazard ratios and 95% confidence intervals were calculated. Statistical significance was set at *p* < 0.05. To forecast the 1-year, 3-year, and 5-year OS, nomograms were constructed using multivariate Cox analysis. The predictive ability of the nomogram was assessed according to the area under the curve (AUC) of the receiver operating characteristic (ROC) curve with better recognition ability, with an AUC closer to 1.0 [11]. The concordance statistic [12] and Brier score [13] of the original and verified models were contrasted through internal validation by bootstrapping (1000 resampling). The overall income under each probable risk threshold was calculated using decision curve analysis (DCA) [14] and the clinical effect of the nomogram was evaluated. The recruited patients were divided into low- and high-risk groups based on the median of the total nomogram scores. Kaplan-Meier analysis [15] was used to estimate survival in the total population, FIGO stage I, II, and III patients. Statistically significant differences between the low- and high-risk groups were analyzed using the log-rank test.

## Results

### Patient characteristics

Based on the standards of inclusion and exclusion, we collected data from the SEER database for 1401 of 1822 MOGCT patients registered between 2004 and 2015. The basic information of the recruited patients are shown in Table 1. The most common demographic characteristics included age less than 27 years (67.24%). The most common clinical characteristics of the patients included: FIGO stage I (68.81%), tumors located on only one side (96.29%), underwent local resection (51.68%) and chemotherapy (57.67%), but no radiotherapy (99.29%), and had histological subtypes of teratocarcinoma (55.03%).

**Table 1:** Characteristics with malignant ovarian germ cell tumor patients

Variables	Training cohort No. (%)	External validation cohort No. (%)	***P***-value
**Total**	**1401**	**121**	
**Age (years)**			0.066
≤ 27	942 (67.24%)	70 (57.85%)	
28–38	304 (21.70%)	37 (30.58%)	
≥ 39	155 (11.06%)	14 (11.57%)	
**Tumor size (mm)**			0.863
≤ 130	533 (38.04%)	47 (38.84%)	
131–175	367 (26.20%)	29 (23.97%)	
≥ 176	501 (35.76%)	45 (37.19%)	
**FIGO Stage**			0.537
FIGO Stage I	964 (68.81%)	81 (66.94%)	
FIGO Stage II	99 (7.07%)	12 (9.92%)	
FIGO Stage III	264 (18.84%)	24 (19.83%)	
FIGO Stage IV	74 (5.28%)	4 (3.31%)	
**Laterality**			0.482
Only one side	1349 (96.29%)	119 (98.35%)	
Both sides	48 (3.43%)	2 (1.65%)	
Unkown	4 (0.29%)	0 (0.00%)	
**Histological subtype**			0.016
Dysgerminoma	405 (28.91%)	38 (31.40%)	
Yolk sac tumor	207 (14.78%)	19 (15.70%)	
Teratocarcinoma	771 (55.03%)	63 (52.07%)	
Non-gestational choriocarcinoma	18 (1.28%)	1 (0.83%)	
**Grade**			0.941
Grade I	120 (8.57%)	9 (7.44%)	
Grade II	172 (12.28%)	16 (13.22%)	
Grade III	216 (15.42%)	18 (14.88%)	
Grade IV	91 (6.50%)	6 (4.96%)	
Unkown	802 (57.24%)	72 (59.50%)	
**Surgery**			0.117
No	13 (0.93%)	3 (2.48%)	
Local resection	724 (51.68%)	69 (57.02%)	
Debulking or cytoreductive surgery or pelvic exenteration	664 (47.39%)	49 (40.50%)	
**Radiation**			0.559
No	1391 (99.29%)	119 (98.35%)	
Yes	10 (0.71%)	2 (1.65%)	
**Chemotherapy**			0.568
No	593 (42.33%)	55 (45.45%)	
Yes	808 (57.67%)	66 (54.55%)	

*FIGO* Federation International of Gynecology and Obstetrics

### Analysis of patient prognosis

The results of the univariate and multivariate Cox regression analyses of factors influencing OS are shown in Table 2. Overall, demographics of older age (≥ 39 years), and clinical parameters of FIGO IV, yolk sac tumor, histology grade IV, and no surgery were linked with an increased risk of death (*P* < 0.05).

**Table 2:** The univariable and multivariate Cox regression analysis of overall survival

Variables	Univariate Cox Regression			Multivariate Cox Regression		
	HR	95%CI	***P*** value	HR	95%CI	***P*** value
**Age (years)**						
≤ 27	Reference			Reference		
28–38	1.42	0.79–2.56	0.249	1.57	0.86–2.88	0.144
≥ 39	7.12	4.45–11.39	< 0.001*	5.70	3.34–9.73	< 0.001*
**Tumor size (mm)**						
≤ 130	Reference			–	–	–
131–175	0.81	0.48–1.39	0.458	–	–	–
≥ 176	0.73	0.45–1.21	0.222	–	–	–
**FIGO Stage**						
FIGO Stage I	Reference			Reference		
FIGO Stage II	2.56	1.05–6.24	0.039*	1.96	0.78–4.90	0.151
FIGO Stage III	4.54	2.68–7.69	< 0.001*	3.93	2.23–6.93	< 0.001*
FIGO Stage IV	16.19	9.24–28.37	< 0.001*	8.11	4.26–15.45	< 0.001*
**Laterality**						
Only one side	Reference			Reference		
Both sides	3.14	1.51–6.50	< 0.001*	1.03	0.46–2.33	0.942
Unkown	14.94	3.66–61.01	< 0.001*	1.19	0.26–5.34	0.825
**Histological subtype**						
Dysgerminoma	Reference			Reference		
Yolk sac tumor	5.41	2.60–11.28	< 0.001*	3.86	1.82–8.19	< 0.001*
Teratocarcinoma	2.36	1.19–4.69	0.014*	2.06	0.98–4.29	0.055
Non-gestational choriocarcinoma	20.82	7.90–54.84	< 0.001*	3.82	1.24–11.75	0.020*
**Grade**						
Grade I	Reference			Reference		
Grade II	8.18	1.06–63.37	0.044*	5.82	0.74–45.69	0.094
Grade III	10.30	1.37–77.12	0.023*	6.06	0.79–46.61	0.084
Grade IV	14.68	1.878–114.67	0.010*	8.41	1.04–67.68	0.045*
Unkown	7.22	1.00–52.32	0.051	4.55	0.61–34.20	0.141
**Surgery**						
No	Reference			Reference		
Local resection	0.04	0.02–0.10	< 0.001*	0.18	0.07–0.49	< 0.001*
Debulking or cytoreductive surgery or pelvic exenteration	0.16	0.07–0.37	< 0.001*	0.25	0.10–0.61	0.002*
**Radiation**						
No	Reference			Reference		
Yes	11.12	4.50–27.51	< 0.001*	2.52	0.89–7.10	0.081
**Chemotherapy**						
No	Reference			–	–	–
Yes	1.29	0.83–2.01	0.256	–	–	–

*HR* Hazard Ratio, *CI* Confidence Interval, *FIGO* Federation International of Gynecology and Obstetrics; *means *p* < 0.05

### Nomogram construction to predict OS

**Fig. 3:**
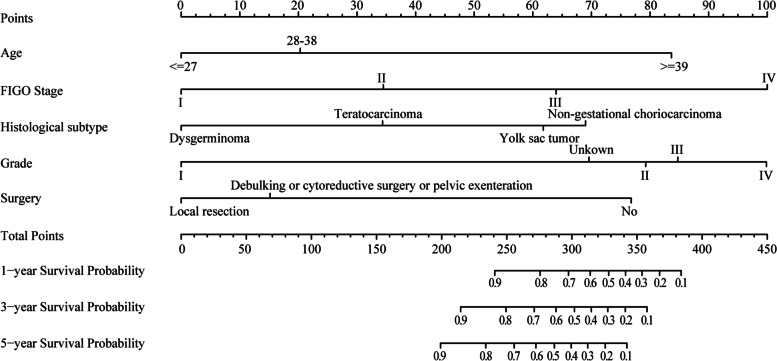
The nomograms of 1-, 3-, and 5-year overall survival (OS)

A nomogram of 1-, 3-, and 5-year OS was constructed using significant variables from the multivariate Cox regression analysis, including age, FIGO stage, histological subtype and grade, as well as the type of surgery. The nomogram revealed that histological grade, FIGO stage, and age had the greatest effect on OS, followed by histological subtype, type of surgery, and ethnicity (Fig. 3).

### Performance of nomogram for assessing OS

**Fig. 4:**
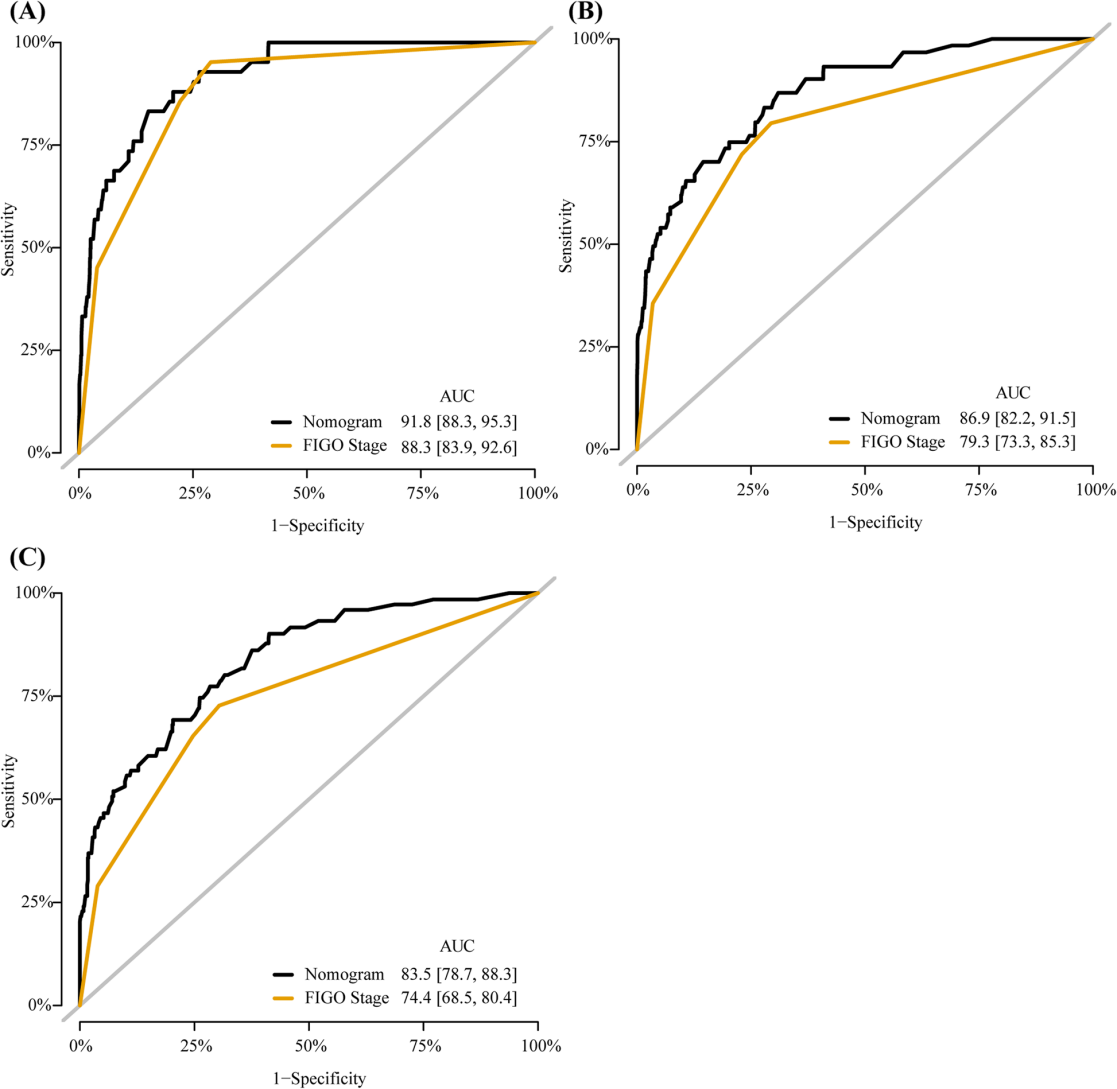
The receiver operating characteristic (ROC) curve for overall survival (OS). **A** ROC curve for 1-year OS; (**B**) ROC curve for 3-year OS; (**C**) ROC curve for 5-year OS

**Fig. 5:**
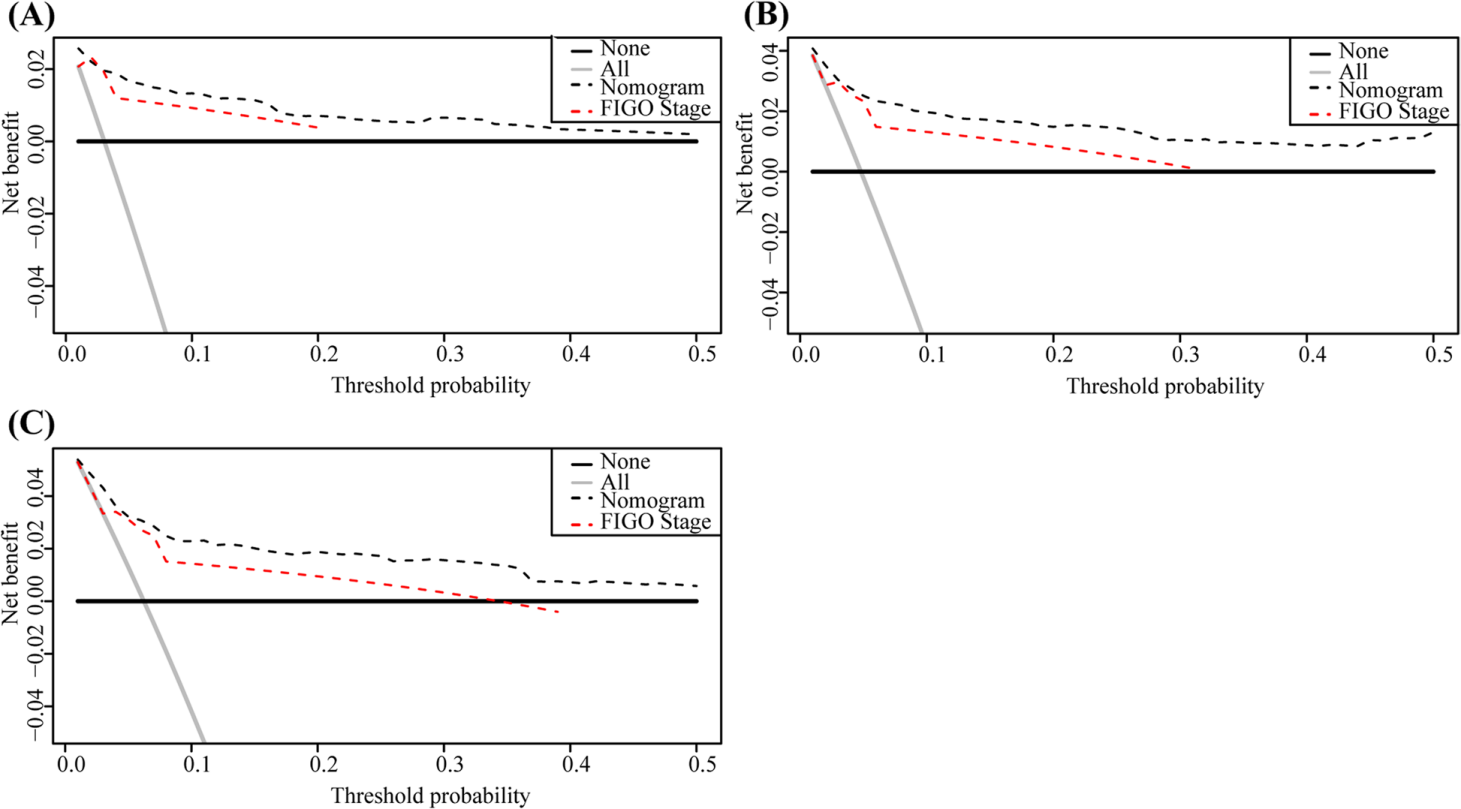
The decision curve analysis (DCA) curve for overall survival (OS). **A** DCA curve for 1-year OS; (**B**) DCA curve for 3-year OS; (**C**) DCA curve for 5-year OS

**Fig. 6:**
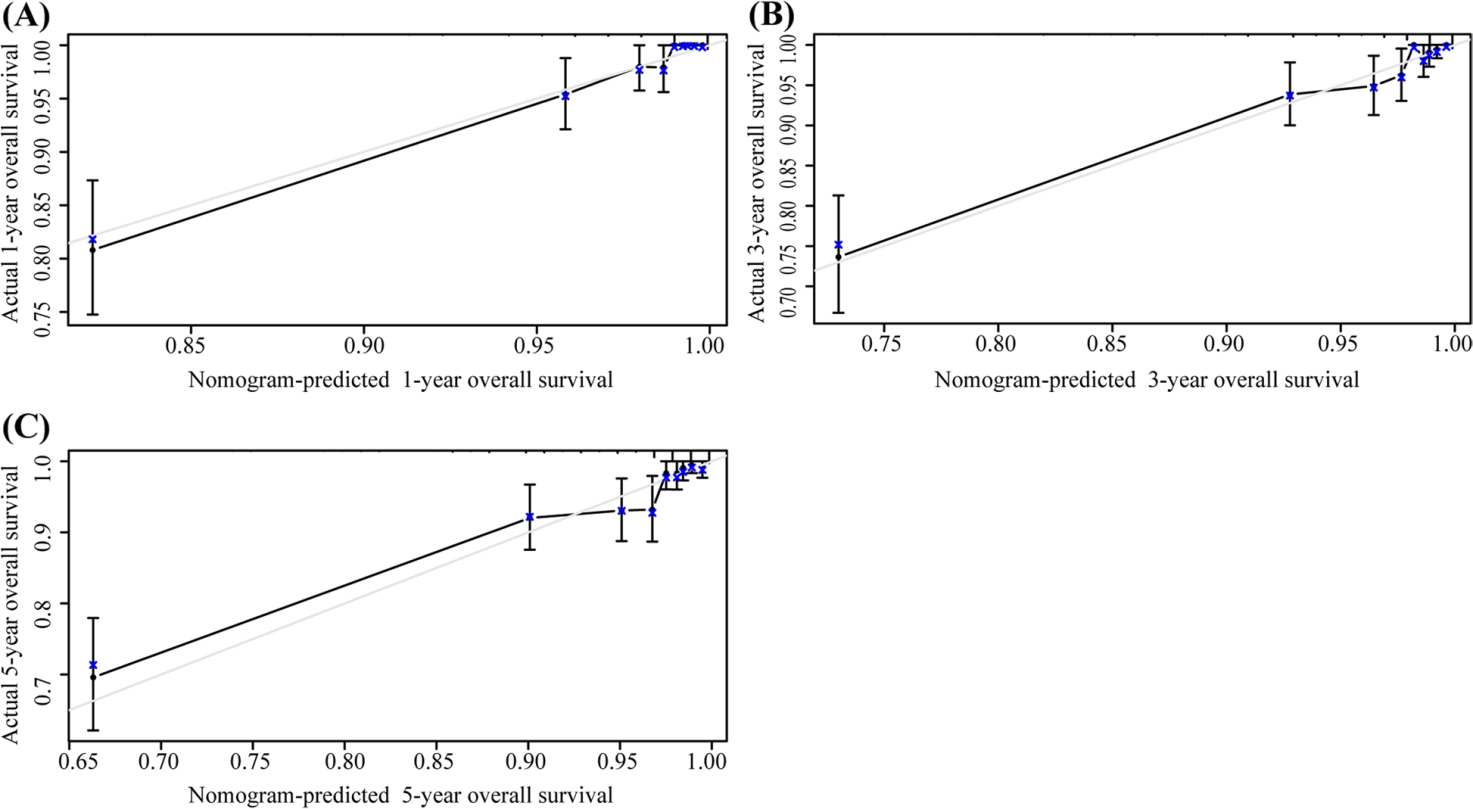
Internal verification plots of nomogram calibration curves by bootstrapping with 1000 resamples. (**A**) 1-year overall survival (OS); (**B**) 3-year OS; (**C**) 5-year OS

**Fig. 7:**
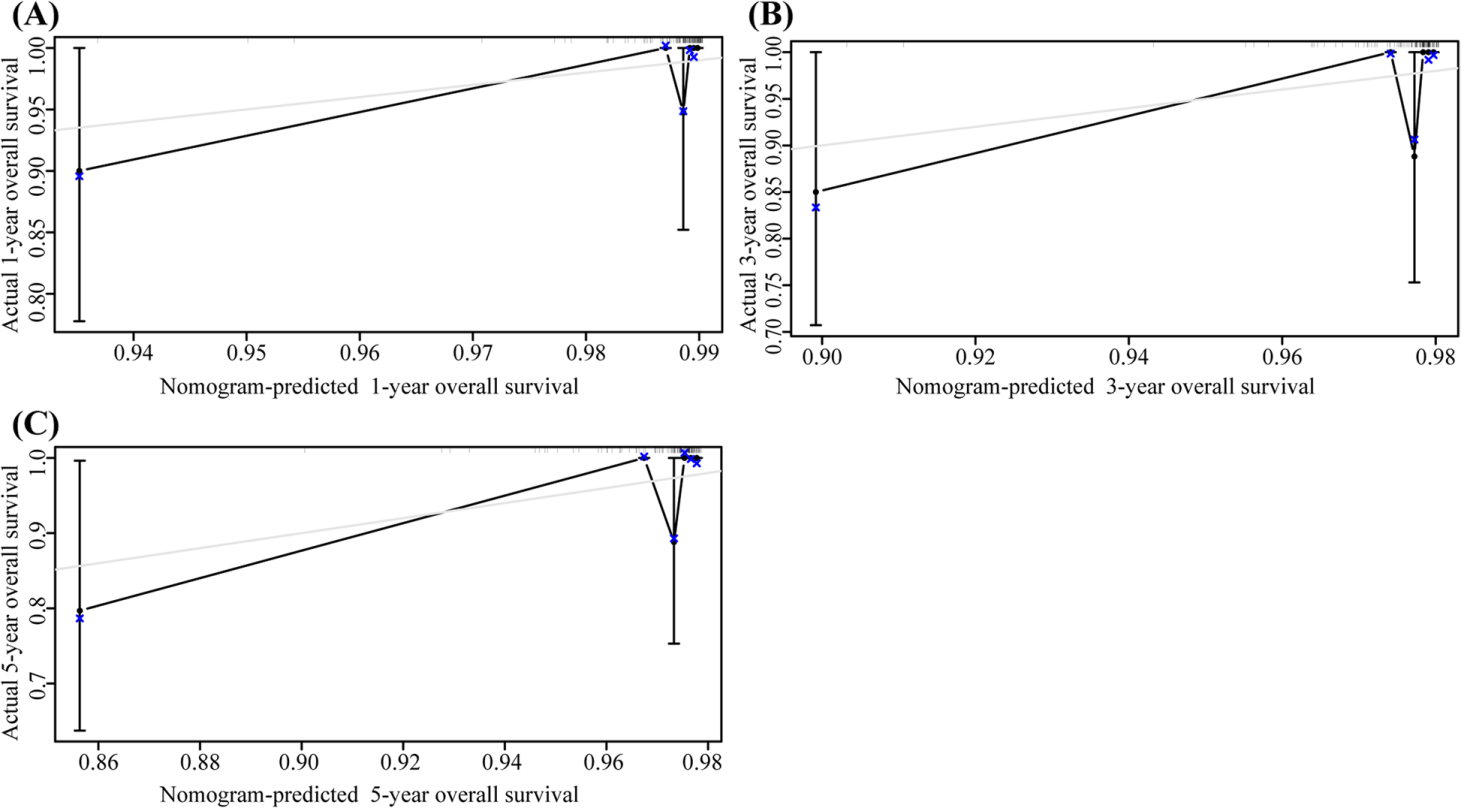
Calibration curve of nomogram in external validation cohort. **A** 1-year overall survival (OS); (**B**) 3-year OS; (**C**) 5-year OS

The nomogram including 1-, 3-, and 5-year OS had an AUC of more than 80% and had a higher predictive power than the nomogram with FIGO staging alone (Fig. 4). The DCA of the nomogram is shown in Fig. 5. The results suggest that the nomogram is more beneficial than the FIGO staging. The calibration curve after internal verification demonstrated that the perceived probability is consistent with the forecast of the nomograms, with all the calibration curves being close to the 45° line (Fig. 6). The Brier scores and C statistics before and after internal verification are presented in Table 3, and further indicate the congruence between the predicted probability and actual probability. The external validation results show that the nomograms were well-calibrated when predicting 1-, 3-, and 5-year OS likelihoods (Fig. 7).

**Table 3:** C-statistic and Breir score of nomograms

Characteristics	1 year	3 year	5 year
**C-statistic**			
Training cohort	0.9181	0.8685	0.8348
After internal verification	0.8944	0.8471	0.8085
After external verification	0.8653	0.8367	0.8593
**Brier score**			
Training cohort	0.0226	0.0333	0.0448
After internal verification	0.0242	0.0353	0.0479
After external verification	0.0242	0.0319	0.0364

### Survival analysis

**Fig. 8:**
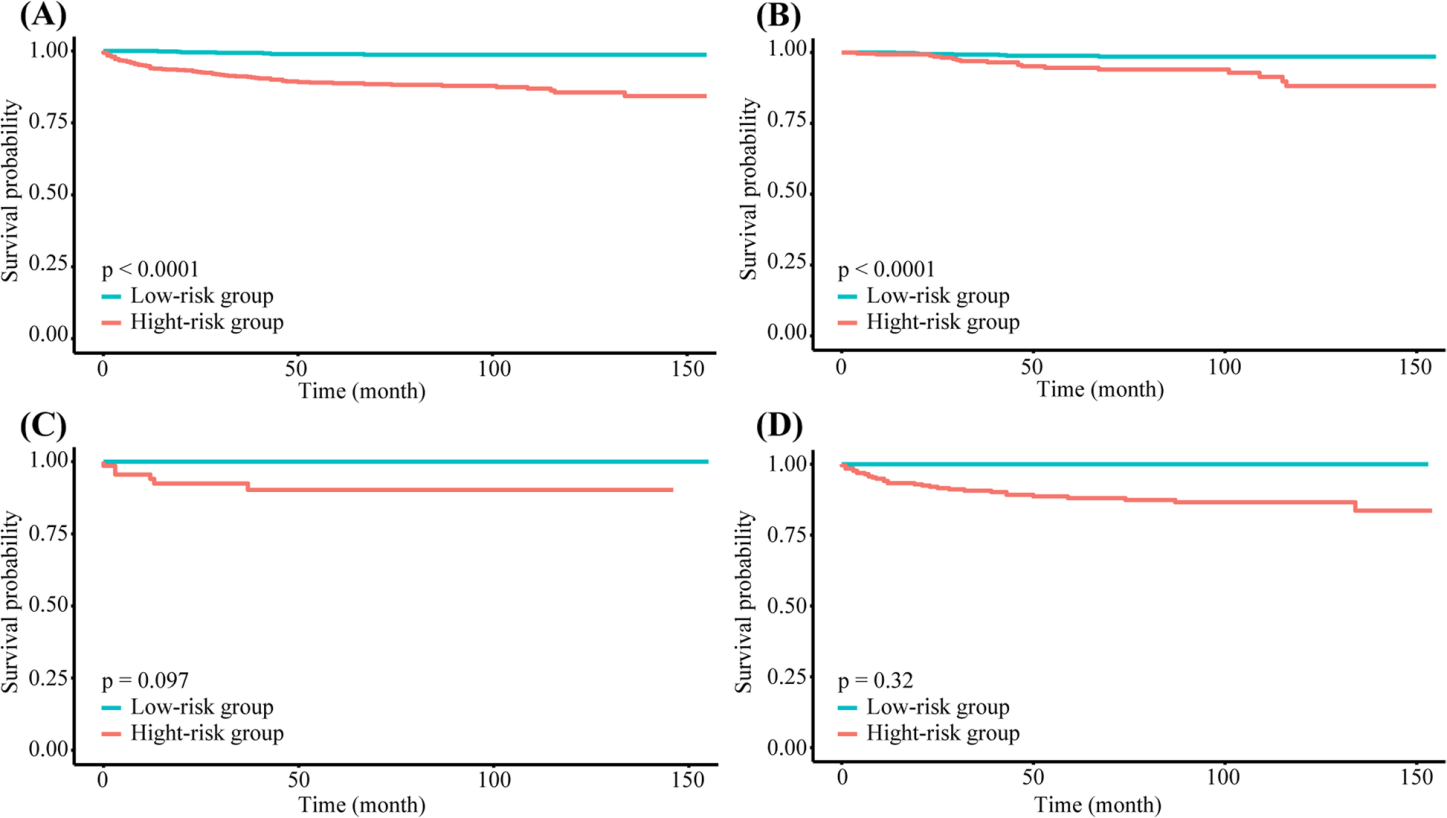
Kaplan-Meier survival curve for malignant ovarian germ cell tumor patients. (A) Overall; (B) FIGO Stage I; (C) FIGO Stage II; (D) FIGO Stage III

Each patient had a calculated prognosis score based on different variables. The median prognosis score (133 points) was adopted as the critical value and was used to categorize patients into low- and high-risk groups. A considerable decrease in OS time was observed in the high-risk group in the general population (*P* < 0.05) and FIGO I patients (*p* < 0.05), indicating that the overall predictive capability of the model was acceptable. The function of the total prognosis score of FIGO II and III was not significant (*P* = 0.097 and *P* = 0.32, respectively), which may have been due to the small sample size of patients within these stages (Fig. 8).

## Discussion

### Main findings

Our study constructed a nomogram of OS for MOGCTs based on the SEER database. The nomogram can better predict OS of MOGCTs, and has better clinical benefits.

### Strengths and limitations

Although our study was the first to generate a nomogram of MOGCT based on data from the SEER database, it has some limitations. First, more than 20% of the potential patients were excluded from the search, possibly because of selection bias. Second, due to limitations of the database, some factors affecting OS, such as molecular markers, were not used in the development of the nomograms [16, 17]. Third, factors such as different doses and durations of chemotherapy were not considered in the model. Finally, the sample size of the external validation queue of this model was small. Future research combining data from other centers to the model may comprehensively improve its validity with regard to predictions.

### Interpretation

Although MOGCTs are depicted as highly malignant, rapidly growing, and large, the survival rate of patients has significantly improved because of the sensitivity of MOGCTs to platinum-based chemotherapy [18, 19]. A combination of tumor resection and platinum chemotherapy results in a five-year survival rate of nearly 90% of patients [20, 21]. However, the prognosis of disease relapse after chemotherapy remains poor, especially in patients with higher grades and higher stages of disease [22], making it important for clinicians to distinguish high-risk factors that influence prognosis. Therefore, the current study aimed to construct a more comprehensive prognostic model to improve the survival of patients with MOGCTs.

Currently, nomograms are widely used as prognostic tools for integrating demographic and clinical characteristics to predict tumor prognosis [23, 24]. However, no previous study has established a nomogram for the prognosis of MOGCT, probably because of the rarity of ovarian germ cell tumors. A nomogram using data available in the SEER database was designed in the present study, which includes clinically useful and readily available parameters, such as age, FIGO stage, histological subtypes, histological grade, and surgical modality. The nomogram has a better predictive power and clinical utility than the simple FIGO staging system using ROC and DCA analyses. Excellent consistency between the predicted and observed OS was observed through internal validation. Based on our findings, nomograms can be used to effectively assess prognoses of MOGCTs and provide individual references for the follow-up treatment of patients.

Due to the high incidence of MOGCT in young women and its sensitivity to platinum-based chemotherapy, it is reasonable to reduce the scope of surgery and preserve fertility, while still improving the cure rate. The effectiveness of comprehensive staging has generated strong deliberations. Hu et al. conducted a retrospective analysis of 137 patients admitted between 1991 and 2014 and found that after adjusting for stage, age, histology, and other risk factors, fertility preservation surgery did not affect the prognosis of patients with MOGCT [25]. Furthermore, in a study of 144 patients with MOGCTs, Mangili et al. showed that fertility preservation surgery was not significantly associated with disease outcomes [26]. However, other studies have contented against this. For instance, Lin et al. demonstrated that comprehensive surgical staging was associated with lower recurrence rates [27]. In the nomogram of our current study, the risk score was significantly increased for patients who have not undergone surgery, and the risk factor scores of debulking or cytoreductive surgery or pelvic exenteration were slightly higher compared to that of local resection. Therefore, surgical treatment is crucial for a positive prognosis. Compared with local resection, expanding the scope of surgery is not very beneficial for prognosis.

Current guidelines for adult women recommend that localized ovarian dysgerminoma and stage I teratocarcinoma require postoperative observation for management. Current guidelines recommend postoperative chemotherapy for all other histologic types, as well as for advanced disease [28]. However, the effectiveness of chemotherapy has been contested. For instance, Billmire et al. observed 56 patients with stage I MOGCT who received chemotherapy and 24 MOGCT patients who did not receive chemotherapy and found that the 5-year OS of both patient groups was 96%, suggesting that most patients are not indicated to undergo postoperative chemotherapy when diseases are diagnosed early [29]. Furthermore, Mangili et al. found no correlation between postoperative chemotherapy and recurrence in patients with teratocarcinoma through univariate analysis [5]. Our current study showed that chemotherapy was not associated with OS in patients with MOGCTs. However, due to the limitations of the SEER database, our study did not include specific chemotherapy regimens or chemotherapy duration. Thus, the results may be limited by bias and are inconclusive for specific chemotherapy regimens.

## Conclusion

In summary, the nomogram of this study demonstrated better prognostic accuracy than that of the FIGO staging system and reliably predicted 1-year, 3-year, and 5-year OS in patients with MOGCTs. This nomogram may prove to be a good predictive tool for gynecological clinical practice and help in the management of patients with MOGCTs.

## Data Availability

Publicly available datasets were analyzed in this study. This data can be found here: https://seer.cancer.gov/data/. Additional data supporting the results of this study are available from Shengjing Hospital of China Medical University, but their availability is limited and they were used with the permission of the current study, so they are not publicly available. However, data can be obtained from the authors upon reasonable request and with the permission of Shengjing Hospital of China Medical University. If anyone would like to obtain data from this study, please contact Zixuan Song, the first author.

## Abbreviations

MOGCTs: Malignant ovarian germ cell tumor
OS: Overall survival
SEER: Surveillance, Epidemiology, and End Results
ICD-O-3: International Classification of Diseases for Oncology, Third Edition
FIGO: Federation International of Gynecology and Obstetrics
AUC: Area under receiver operating characteristic curves
ROC: Receiver operating characteristic
DCA: Decision curve analysis
HR: Hazard ratios
CI: Confidence intervals
